# Isolation and screening of a chitin deacetylase producing *Bacillus cereus* and its potential for chitosan preparation

**DOI:** 10.3389/fbioe.2023.1183333

**Published:** 2023-03-30

**Authors:** Yingying Zhang, Xi Luo, Longfei Yin, Fengwei Yin, Weilong Zheng, Yongqian Fu

**Affiliations:** Taizhou Key Laboratory of Biomass Functional Materials Development and Application, Taizhou University, Taizhou, Zhejiang, China

**Keywords:** chitin deacetylase, chitosan, *Bacillus* cereus, degree of deacetylation, green preparation

## Abstract

Chitosan is a biopolymer material extracted from marine biomass waste such as shrimp and crab shells, which has good biocompatibility and degradability with great potential for application in the field of wastewater treatment and soil remediation. The higher the degree of deacetylation (DD), the better the adsorption performance of chitosan. Chitin deacetylase (CDA) can specifically catalyze the deacetylate of chitin in a green reaction that is environmentally friendly. However, the scarcity of high yielding chitin deacetylase strains has been regarded as the technical bottleneck of chitosan green production. Here, we screened a natural chitin degrading bacterium from coastal mud and identified it as *Bacillus cereus* ZWT-08 by re-screening the chitin deacetylase activity and degree of deacetylation values. By optimizing the medium conditions and enzyme production process, ZWT-08 was cultured in fermentation medium with 1% (m/V) glucose and yeast extract at pH 6.0, 37°C, and a stirring speed of 180 r/min. After fermenting in 5 L fermenter for 48 h, the deacetylation activity of the supernatant reached 613.25 U/mL. Electron microscopic examination of the chitin substrate in the fermentation medium revealed a marshmallow-like fluffy texture on its structural surface. Meanwhile, 89.29% of the acetyl groups in this chitin substrate were removed by enzymatic digestion of chitin deacetylase produced by ZWT-08, resulting in the preparation of chitosan a degree of deacetylation higher than 90%. As an effective strain for chitosan production, *Bacillus cereus* ZWT-08 plays a positive role in the bioconversion of chitin and the upgrading of the chitosan industry.

## 1 Introduction

Soil and water contamination incidents occurred frequently in recent years. Research has found that the use of biomass materials for environmental remediation is one of the most efficient and economical techniques to achieve the recycling of resources and less damage to the environment (Lan et al., 2021; Chakraborty et al., 2022). Chitosan (β-(1–4)-2-amino-2-deoxy-D-glucan), also known as deacetylated chitin, is the only alkaline polysaccharide among natural polysaccharides and is safe, bioadhesive, and degradable (Aranaz et al., 2021; Kou et al., 2022). As a result, it has been widely used in wastewater treatment and soil remediation. (Pal et al., 2020; Hu et al., 2021; Keshvardoostchokami et al., 2021).

Industrial wastewater containing heavy metal ions is often highly toxic and difficult to degrade, causing great pollution to the environment. Chitosan molecules contain a variety of active groups such as hydroxyl (-OH), amino (-NH_2_), and amide groups (-CONH_2_-) (Ke et al., 2021). Recent studies suggest chitosan microspheres prepared by ion exchange, emulsification crosslinking, or precipitation condensation can effectively adsorb heavy metal ions in water (Yu et al., 2017; Ren et al., 2019; Liu et al., 2022). A knowledge of -NH_2_ root is the characteristic functional group for the adsorption of metal ions (Ahmad et al., 2017; Ke et al., 2021). The higher the degree of deacetylation (DD) of chitosan, the higher the content of -NH_2_ root, and the better its adsorption performance (Jóźwiak et al., 2017). For example, chitosan synthesized with a higher deacetylation degree and lower molecular weight has shown good adsorption properties of heavy metals and antibiotics (levofloxacin and tetracycline hydrochloride) (Jiang et al., 2021).

The traditional preparation method of chitosan is the chemical degradation of shrimp skin and crab shells. However, the use of strong acids and alkalis leads to high energy consumption, unstable product properties, and great pollution to the environment (Tokatli and Demirdöven, 2018; Al Hoqani et al., 2020; Kou et al., 2021). Chitin deacetylase (CDA) is a metalloenzyme that specifically deacetylates chitin with a green reaction process, stable product quality and uniform acetylation (Chakraborty et al., 2016; Yang et al., 2022). The microbial sources of CDAs are narrow, with fungi such as *Absidia coerulea, Aspergillus nidulans*, and *Penicillium oxalicum* as the main ones, and bacteria such as *Bacillus* and *Vibrio* (Win and Stevens, 2001; Liu et al., 2017; Liang et al., 2022). Presently, there is no industrial method in existence for generating this enzyme, primarily due to factors including insufficiency of promising microbial varieties and intricate fermentation prerequisites for chitin deacetylase output in numerous reported strains (Suresh et al., 2014). Therefore, there is an urgent need to screen for new strains with excellent CDA production.

Here, a strain capable of degrading natural chitin was isolated from coastal mud and re-screened for enzyme activity and chitin deacetylation. The strain was used for liquid fermentation to produce CDA, and its medium conditions and production process were optimized. Further scale-up experiments were carried out using the fermenter to provide a reference for the industrial production of CDA.

## 2 Materials and methods

### 2.1 Bacterial isolation

Activated silt samples were collected from the coastal intertidal zone of Zhejiang Province, China. Microbial consortia were obtained by enrichment culture with chitin medium and 1 g of silt per 60 mL of the medium as inoculum. The chitin medium contained per liter of deionized water: 20 g Chitin (DD 8%, Golden-Shell Pharmaceutical Co., Ltd., Zhejiang, China), 0.5 g NaCl, 2.31 g KH_2_PO_4_, 12.54 g K_2_HPO_4_, 0.5 g MgSO_4_·7H_2_O, 0.01 g Fe_2_(SO_4_)_3_. 4-Nitroacetanilide (Sinopharm Chemical Reagent Co., Ltd., Shanghai, China) (at a concentration of 10 g/L) was then added as the nitrogen source. Congo red (at a concentration of 1 g/L) was added as an indicator. Enrichments and isolates were grown in an incubator at 37°C for 3–4 days. A yellow chromogenic circle appeared around the CDA-producing strain, whose shade represents the enzyme-producing capacity of the strain (Zhou et al., 2010). Individual isolates were obtained by plating 10-fold serial dilutions of the enrichments on selective chitin plates, transferred to chitin media plus peptone (at a concentration of 10 g/L) plates, and then isolated by streaking on chitin media plus 4-Nitroacetanilide plates until pure.

The screened strain was cultivated in a 250 mL Erlenmeyer flask containing 60 mL of the fermentation medium (chitin media plus 10 g/L peptone) at 37°C with shaking at 180 rpm for 48 h. The fermentation broth was centrifuged at 9,000 × g for 5 min at 4°C, and the supernatant obtained was the crude enzyme solution of extracellular CDA enzyme of which the activity was determined by enzyme activity assays. The bottom moist solid remaining after centrifugation was used to determine the DD value. According to the enzyme activity and DD level, the strains with a higher ability to deacetylate chitin were selected.

### 2.2 Enzyme activity assay

Enzyme activity assay was performed using the method described previously (Liu et al., 2016) with slight modifications. The properly diluted supernatant (1 mL) was incubated with 1 mL 4-Nitroacetanilide (200 mg/L) in phosphate buffer (0.05 M, pH 7.2) at 50°C for 20 min. The reaction was terminated by heating at 100°C for 3 min. After cooling to room temperature, the reaction system was filled to 10 mL by adding distilled water, mixed, and then centrifuged at 10,000 × g for 5 min. The absorbance value (*OD*_400_) of the supernatant was measured. Heat-inactivated enzyme solution was used as a control group. The standard curve of p-Nitroaniline was measured according to the *OD*_400_ values of serially diluted p-Nitroaniline (Sinopharm Chemical Reagent Co., Ltd., Shanghai, China) samples. A unit of enzyme activity was defined as the amount of enzyme required to produce 1 μg of p-Nitroaniline per hour under the above reaction conditions.

### 2.3 Deacetylation degree determination

The DD values of chitosan were determined by an acid-base conductometric titration method (Tan et al., 2012; Dutta and Priyanka, 2022). The bottom moist solid after centrifugation of the fermentation broth was dried at 40°C. The dried solid (0.2 g) was dissolved in 20 mL of 0.1 mol/L HCl solution. Then, 2-3 drops of methyl orange (1%, m/V) were added as the indicator. The mixture was stirred at room temperature with a magnetic stirrer for 0.5–1 h until dissolved. Titrate the excess HCl with 0.1 mol/L NaOH standard solution until the color changes from pink to yellow orange which means the end point of titration. Based on the volume of NaOH used at the end of titration, the DD value of chitosan was calculated using the following equation according to Dutta and Priyanka (Dutta and Priyanka, 2022):

$$\%\;ofDD=\frac{\left(V1-V2\right)\times 16}{V1\times 9.94\times x}\times 100$$

Where, *V1* is the volume of chitosan solution prepared in 0.1 mol/L HCl solution in mL; *V2* is the volume of 0.1 mol/L NaOH in mL; 16 is the gram equivalent weight of -NH_2_; 9.94 is the theoretical value of % NH_2_ group content of chitosan; x is the weight of dried chitosan.

### 2.4 Bacterial identification

The screened strain was identified based on colony morphology, gram staining, and spore staining. Molecular identification was performed by amplification of 16S rDNA with bacterial universal primers 27F and 1492R. Total genomic DNA was extracted from the selected isolates by a rapid bacterial genomic DNA isolation kit (Sangon Biotech, Shanghai, China). Amplifications were performed in a 25 μL reaction mixture containing 1 μL of genomic DNA, 1 μL of each primer, 2.5 μL 10×PCR Buffer, 2 μL of dNTP, and 0.5 μL of *Taq* polymerase (Takara, Beijing, China). Samples were amplified by 30 cycles consisting of 94°C for 30 s, 54°C for 45 s, and 72°C for 50 s, followed by a final extension step of 10 min at 72°C. The PCR product was purified by the kit and sequenced by Sangon Biotech Co., Ltd. (Shanghai, China). Acquired sequences were compared with the GenBank database by BLASTN to determine the homology of 16 s rDNA gene sequences.

### 2.5 Culture optimization

The optimization of culture medium conditions was performed by substituting components of the basal fermentation medium. The effect of carbon sources on CDA production was investigated by adding the following carbon sources: sucrose, lactose, glucose, maltose, and soluble starch individually to the basal fermentation medium at a 1% (m/V) concentration. To test the effect of nitrogen sources on CDA production, the following nitrogen sources: Beef extract, yeast extract, urea, ammonium sulfate, and sodium nitrate were individually added to substitute the peptone in fermentation medium at a 1% (m/V) concentration. The effects of pH and temperature on CDA production were studied in the optimized culture medium. The effect of pH was determined by adjusting the pH with the phosphate buffer to a range of pH values (pH 5.0–8.0). The influence of temperature was assessed by incubating the shake flasks at 16, 28, 30, 35, 40, and 45°C. The CDA activity determined in the basal fermentation medium at pH 7.0°C and 37°C with shaking at 180 rpm for 48 h was designated as 100% while others were expressed against this value. All experiments were repeated in three parallel.

### 2.6 Growth studies

Growth studies were conducted in 5 L fermenters. *Bacillus cereus* ZWT-08 was grown on the optimized fermentation medium containing glucose and yeast extract at a 1% (m/V) concentration. Fermentation lasted for 60 h with a liquid volume of 3 L, inoculum volume of 10% (m/V), pH 6.0, 37°C, and stirring speed of 180 r/min. By sampling at a time interval of 6 h, the cell wet weight was recorded and the activity of CDA was determined. The reduced sugars were measured according to the DNS method as previously described (Miller et al., 1960).

### 2.7 SEM analysis

The surface structures of chitosan prepared by *Bacillus cereus* ZWT-08 were observed under scanning electron microscopy (SEM) as described below. Samples were washed with ethyl alcohol at room temperature and dried to coat on a double sided conducting adhesive tape. SEM analysis was employed using Talos™ F200i from Thermo Scientific. The images were taken at 10–20 kV.

## 3 Results and discussion

### 3.1 CDA-producing bacteria isolations

**FIGURE 1:**
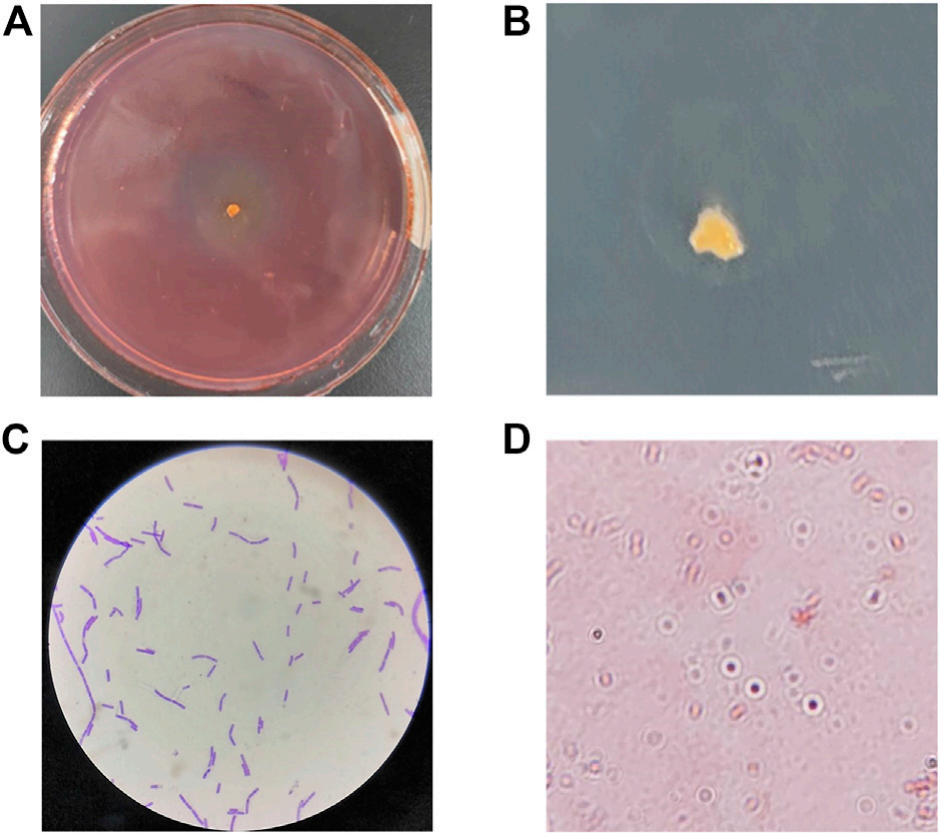
Morphological identification and staining results of *Bacillus cereus* ZWT-08. **(A)** A yellow chromogenic circle appeared around the ZWT-08 in the selective chitin plates, whose shade represents the CDA-producing capacity of the strain. **(B)** Single colony morphology of ZWT-08 in the chitin plates indicated it to be an irregular colony, soft texture, molten wax-like, and no pigment. **(C)** Gram staining results were positive. **(D)** The spore staining was positive.

The marine environment is extremely diverse and marine microorganisms are affected by temperature, salinity stress and nutrient limitation, and thus may have specific metabolic pathways and their secreted enzymes may have a range of quite different biochemical and physiological characteristics. Here we isolated the CDA-producing bacteria from the coastal intertidal zone of Zhejiang Province, China. The objective trains were screened based on their diacetyl ability to remove the acetyl group from 4-Nitroacetanilide to produce the p-nitroanilide, which is yellow in color. Therefore, the shade of color and the size of color-forming circles were the basis of the screening of high enzyme-producing strains. Combined with the spectrophotometric method to determine the CDA activity, the screening results were obvious. A total of 7 CDA-producing bacteria were isolated from the active sludge samples, namely, ZWT-01, ZWT-08, S-03, BD-05, BD-10, ZY-03, and ZY-07. The isolated strains were re-screened by determination of CDA activities and the DD values, of which the highest CDA-producing potential, ZWT-08, was obtained (Table 1). The strain ZWT-08 was stored in China General Microbiological Culture Collection Center (CGMCC No. 22932). Morphological identification and staining results indicated it to be a Gram-positive, spore-producing, irregular colony, soft texture, molten wax-like, and no pigment (Figure 1).

**TABLE 1:** Extracellular CDA activity and DD values of different strains in re-screen.

Bacteria	Extracellular activity (U/mL)	DD values
ZWT-01	79.45	84.52
ZWT-08	135.52	90.15
S-03	48.91	47.29
BD-05	64.07	59.60
BD-10	23.58	56.75
ZY-03	90.44	84.36
ZY-07	35.69	55.09

### 3.2 Molecular identification of ZWT-08

The molecular identification was performed based on homology analysis of the 16s rRNA gene sequences. The sequences of strain ZWT-08 were blasted in GenBank for comparison with other reported 16S rDNA sequences. As a result, the 16S rDNA sequence of ZWT-08 was highly homologous to *Bacillus cereus* with a homology of >99%. Based on its strain morphology, physiological and biochemical characteristics, and 16S rDNA sequence homology, strain ZWT-08 was identified as *Bacillus cereus*.

*Bacillus cereus* is a facultative anaerobic *bacillus* widely distributed in nature. Previous studies have shown that *Bacillus cereus* can secrete a variety of antibacterial substances to inhibit the reproduction of harmful microorganisms, degrade nutrients in the soil, and improve the ecological environment (Ming et al., 2022). By standard procedures, Sherin et al. isolated a *Bacillus cereus* strain JCM44 from coastal areas of South India, which can be utilized as a novel source for the prompt production of CDA (Sherin and Arulselvi, 2020). However, the research on the potential CDA production of *Bacillus cereus* was deficient. The present study was designed to obtain useful information from the CDA-producing of *Bacillus cereus* ZWT-08, to provide a new idea for the green preparation of chitosan.

### 3.3 Optimization of CDA production

**FIGURE 2:**
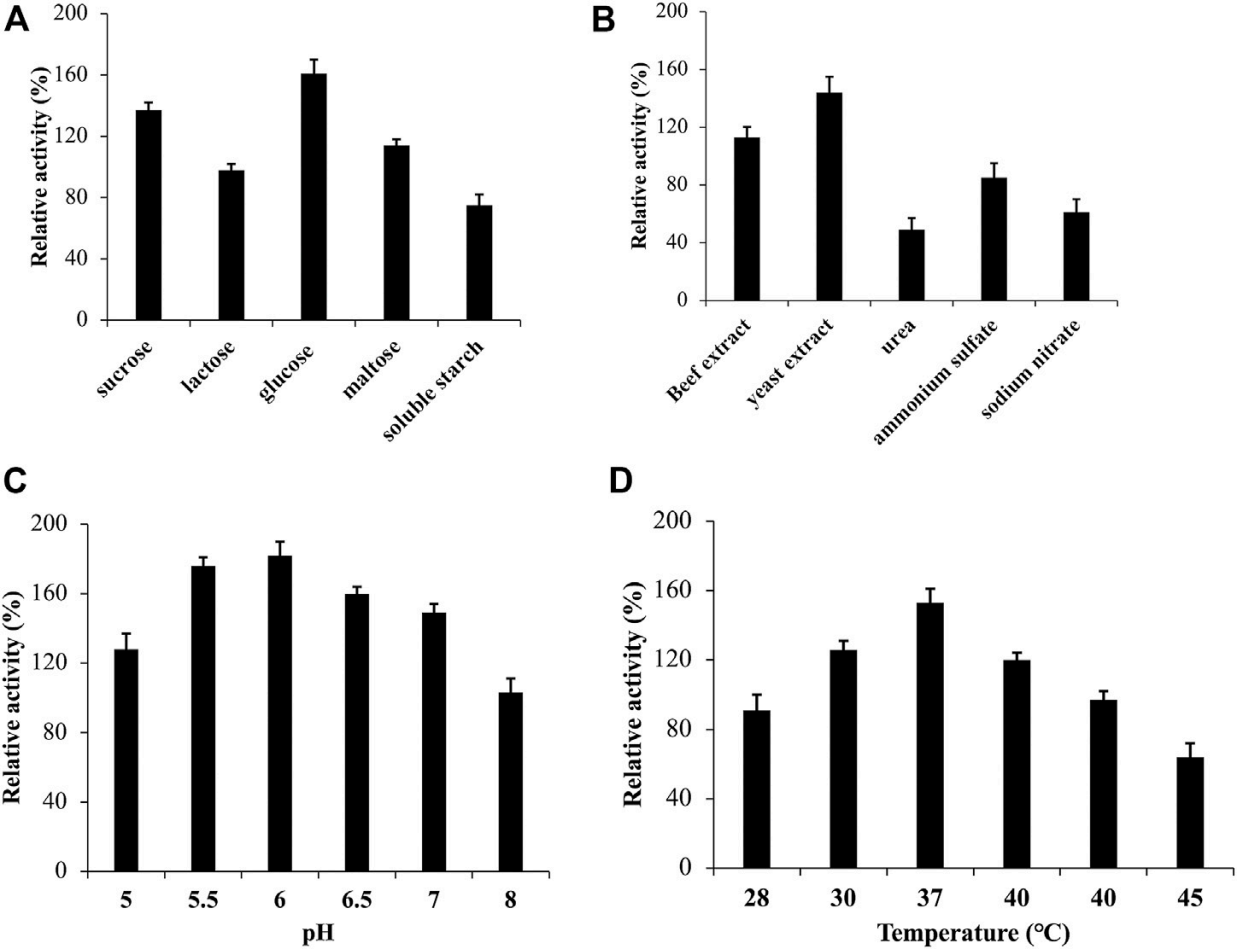
Effect of medium and culture conditions on the CDA activity of *Bacillus cereus* ZWT-08. The effect of carbon sources **(A)** and nitrogen sources **(B)** on the activity of CDA was compared at a concentration of 1% (m/V). The effect of pH **(C)** and temperature **(D)** on the CDA activity was studied in the optimized culture medium. The CDA activity determined in the basal fermentation medium at pH 7.0°C and 37°C with shaking at 180 rpm for 48 h was designated as 100% while others were expressed against this value.

The external environment has a significant impact on the growth of microorganisms. To optimize the medium for strain ZWT-08, a fermentation medium was used, and the CDA activity peaked at 135.52 U/mL after 48 h of culture at pH 7.0°C and 37°C with shaking at 180 rpm, which served as a reference at 100%. As shown in Figure 2A, the relative activity of CDA was 161% when strain ZWT-08 was inoculated in the fermentation medium supplemented with glucose. Sucrose and maltose were also effective carbon sources for CDA production, while the use of soluble starch resulted in a decrease in CDA production of ZWT-08, reducing the relative activity to 75%. The effect of different carbon and nitrogen sources on CDA production was statistically significant (*p* < 0.05) by one-way ANOVA. In Figure 2B, it was observed that the enzyme production capacity of the strain decreased significantly in the presence of inorganic nitrogen sources such as ammonium sulfate and sodium nitrate, compared to organic nitrogen sources like beef extract, yeast extract, and peptone. Organic nitrogen sources were found to have a promoting effect on the synthesis of CDA from ZWT-08, with yeast extract showing the most significant impact, with a relative activity increase of up to 144%.

Based on the optimized fermentation medium, the culture conditions of strain ZWT-08 were optimized. As shown in Figure 2C, the CDA activity increased as pH increased from 5.0 to 6.0. The highest relative CDA production (182%) was achieved at pH 6.0, but declined sharply to 103% at pH 8.0, indicating that the strain ZWT-08 prefers an acidic environment for optimal CDA production. The incubation temperature had a significant effect on the strain, as it can affect microbe growth and metabolite synthesis, including enzymatic inactivation at high temperatures. The optimum temperature for CDA production from strain ZWT-08 was found to be 37°C, with a relative activity of 153% (Figure 2D). According to the findings, the most effective supplementary carbon source for the primary source chitin was glucose, with a concentration of 1%. Similarly, the optimum nitrogen source was 1% yeast extract. Furthermore, the optimal conditions for CDA production were determined to be a pH of 6.0 and a temperature of 37°C.

### 3.4 Batch fermentation of *Bacillus cereus* ZWT-08

**FIGURE 3:**
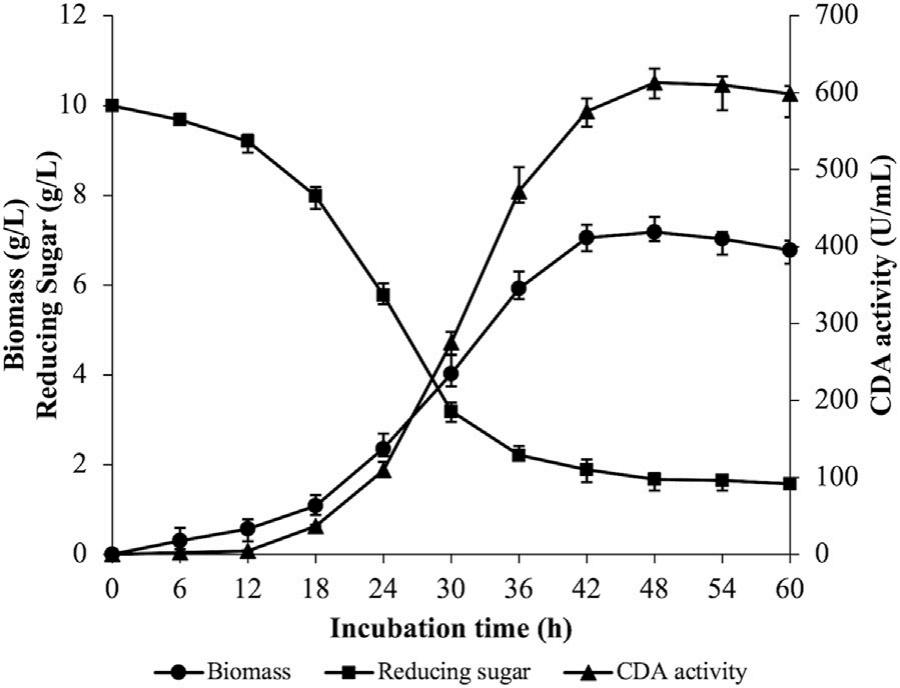
Time courses of biomass content, CDA activity, and reducing sugar in the fermentation broth. The strain ZWT-08 was cultured in a chitin medium containing 1% (m/V) glucose and 1% (m/V) yeast extract at a pH of 6.0, a temperature of 37°C in 5 L fermenters. Error bars represent ± standard deviations (*n* = 3).

A 5 L fermenter was utilized to perform batch fermentation of strain ZWT-08 based on the optimization of CDA production conditions. Figure 3 illustrates the results where the rate of reducing sugar consumption experienced a rapid increase from 12 to 36 h, then gradually decreased with residual reducing sugar concentration at the end of the fermentation being (1.57 ± 0.09) g/L. Biomass peaked at 48 h, where the wet weight of cells was (7.18 ± 0.26) g/L, but declined as the fermentation progressed. The bacterium exhibited logarithmic growth from 18 to 42 h, then showed autolysis with the extension of fermentation time. Enzyme production of strain ZWT-08 increased steadily with the growth of the bacterium density, with extracellular CDA enzyme activity reaching 613.25 U/mL at 48 h. Enzyme activity decreased as the incubation time was extended, possibly due to changes in nutrient composition, stable growth phase of the bacterium followed by decay, enzyme product accumulation, and pH changes in the fermentation system.

**FIGURE 4:**
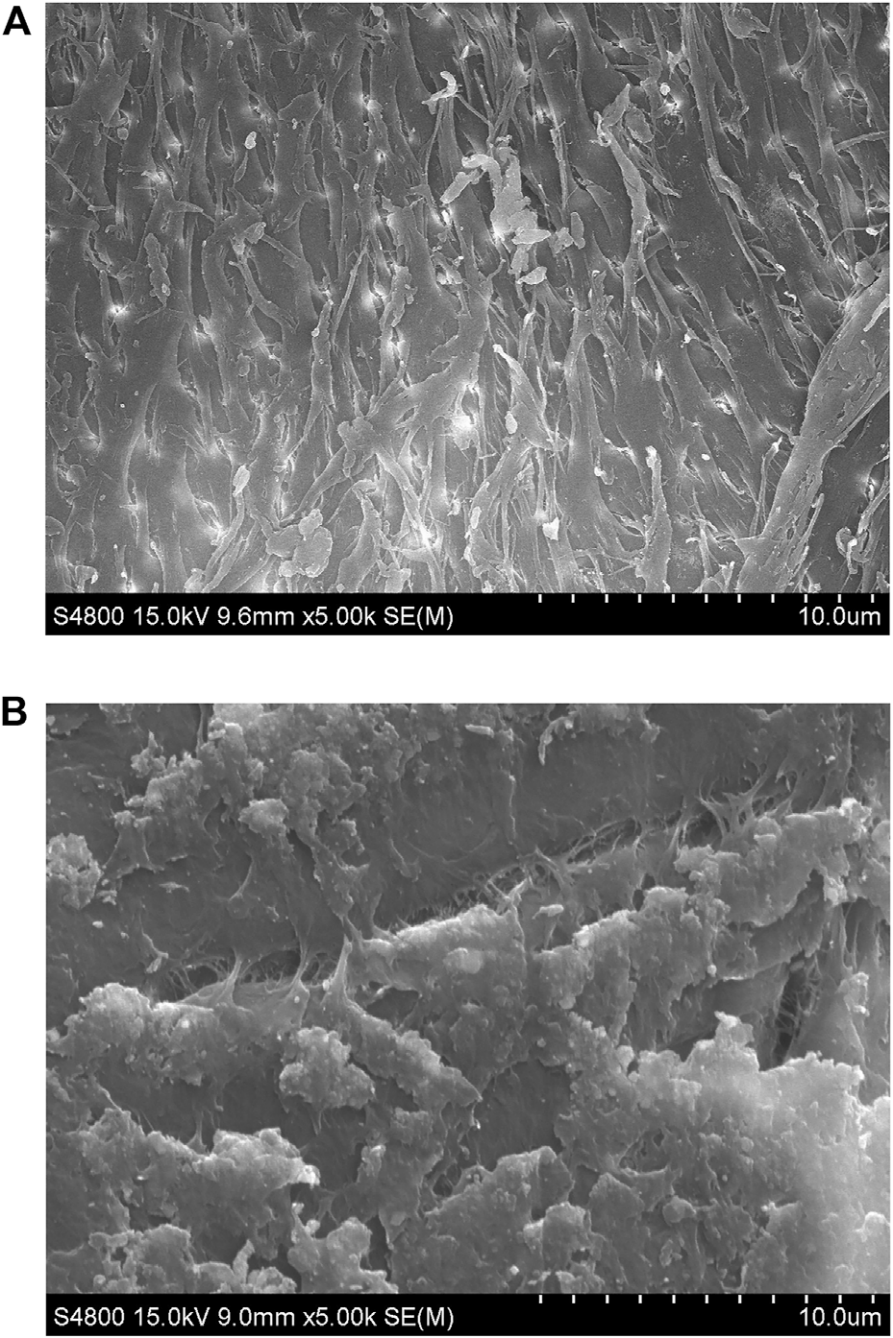
SEM of chitin and chitosan surface structures at ×1,000 magnification. **(A)** Untreated chitin. **(B)** The treated chitin after cultivation with *B. cereus* ZWT-08 for 48 h, which has been deacetylated to chitosan.

The surface structure changes of chitin substrate (8% deacetylation) in the medium before and after fermentation were compared using scanning electron microscopy. After 48 h of fermentation, chitin was observed with significant alterations catalyzed by the enzyme secreted from *Bacillus cereus* ZWT-08. As shown in Figure 4, the resulting product exhibited a marshmallow-like texture and fluffy surface. The DD measurement of the product chitosan showed that it had removed 89.29% of the acetyl group and the DD value reached 90.15%. As we known, this is the strongest deacetylation ability of CDA in bacteria.

Biocatalysis using CDA is an eco-friendlier and more controlled method than conventional chemical approaches. However, the majority of CDA-producing strains show low activity and production. Thus, the enhancement of CDA production has always been a challenge. At present, the best-reported CDA-producing strain is *Rhodococcus equi* CGMCC14861 in co-culture with *Staphylococcus* sp. MC7, the maximum activity of CDA can peak 2,974.05 U/mL (Ma et al., 2020). Unfortunately, there are no relevant studies on the enzymatic characteristics of this CDA. Compared with the co-cultured strains, the CDA production ability of *Bacillus cereus* ZWT-08 isn’t in ascendancy. Nevertheless, the DD of the product obtained by its enzymatic catalysis is the highest reported so far.

In this paper, we studied the fermentation enzyme production of *Bacillus cereus* ZWT-08 with natural chitin used as the substrate. Adding glucose and yeast extract as supplementary carbon sources and sole nitrogen sources, respectively, could rapidly enhance the enzyme production activity of ZWT-08. Through the 5 L fermenter amplification culture, the strain could produce up to 613.25 U/mL of extracellular CDA activity while effectively removing the acetyl group of chitin substrate to prepare chitosan with a DD value higher than 90%, thus realizing the green preparation of chitosan. Based on the results of this study, genetic engineering modification and enzymatic characterization of this strain can be considered at a later stage to construct an engineered strain for the industrial production of chitosan to improve the CDA yield and shorten the fermentation cycle.

## Data Availability

The datasets presented in this study can be found in online repositories. The names of the repository/repositories and accession number(s) can be found below: https://www.ncbi.nlm.nih.gov/genbank/, OQ659020.
